# 4276 Validation of Ototoxicity Prediction Model for Patients with Head and Neck Cancer

**DOI:** 10.1017/cts.2020.446

**Published:** 2020-07-29

**Authors:** Brian Deutsch, Dorina Kallogjeri, Jay Piccirillo

**Affiliations:** 1 Washington University in St. Louis, Institute of Clinical and Translational Sciences

## Abstract

OBJECTIVES/GOALS: To validate the previously developed ototoxicity prediction model for objective (i.e., audiometric-defined) hearing loss from cisplatin-based and radiation treatments in a new cohort of head and neck cancer patients treated from 2018 to 2019. METHODS/STUDY POPULATION: This study will use a cohort of 106 patients undergoing treatment for head and neck cancers at a single institution to temporally validate a model for post-treatment ototoxicity. We are interested in understanding if this model will be able to predict ototoxic risk (calibration) and if this model can differentiate high- and low-risk patients (discrimination). Observed and predicted values for audiometric hearing loss will be calculated and then compared using a calibration curve available in SAS v9.4, while the c-index (area under the receiver-operator curve) will be used to assess discrimination. The implementation of this model will be assessed in a clinical setting. RESULTS/ANTICIPATED RESULTS: The validation cohort is generally similar in age (61 years) and sex-mix (23% female) to the original cohort. However, there seems to be a different case-mix the types of treatments with more patients receiving cisplatin overall (59% vs. 43%), but fewer getting induction and high-dose cisplatin (1% vs. 13%). The original model showed good calibration and fair discrimination in the validation cohort with and area under the curve of 0.700. This concordance statistic suggests possibly-useful discrimination and the calibration curve suggests the model is well-calibrated. DISCUSSION/SIGNIFICANCE OF IMPACT: This project can improve clinical treatment paradigms, enhance patient education, and reduce healthcare costs. Our model allows oncologists to weigh the risks of hearing loss with the benefits of treatment on an individualized level *before* treatment, facilitating informed treatment decision-making.

